# Supplementary material for: A Likelihood Approach for Real-Time Calibration of Stochastic Compartmental Epidemic Models
Source: PLoS Comput Biol. 2017 Jan 17;13(1):e1005257. doi: 10.1371/journal.pcbi.1005257 (PMC5240920; doi:10.1371/journal.pcbi.1005257)
Supplement: S1 File — (TAR.GZ) [file pcbi.1005257.s014.tar.gz › HSPH_Online-SI-Revision/Additions/EnKF08/n100-extreme-SEITR/n100-extreme-SEITR_PF_EnKF-summery.pdf]

peak -8	R0	$1/\theta_2$	inf	inflog	newly	newlylog	Reff	AttackRate
mean	43.8	55.1	73 430.9	2.1	106 821.	2.2	55.8	57.
median	38.8	54.1	19 233.2	2.1	25 379.9	2.2	54.8	55.3
Quantile .1	19.3	38.1	429.9	0.5	821.3	0.6	36.5	31.9
Quantile .9	63.8	70.5	179 308.	3.1	361 842.	3.2	74.3	89.6
43	27	36						
length	50							

peak -4	R0	$1/\theta_2$	inf	inflog	newly	newlylog	Reff	AttackRate
mean	45.1	59.5	490.2	0.7	381.	0.7	70.	80.1
median	37.1	62.5	94.2	0.7	134.1	0.6	71.8	80.4
Quantile .1	18.9	42.2	69.2	0.4	79.3	0.4	53.7	61.2
Quantile .9	84.8	73.8	1328.5	1.	1010.9	0.9	84.2	100.
40	19	20						
length	50							

peak	R0	$1/\theta_2$	inf	inflog	newly	newlylog	Reff	AttackRate
mean	45.8	49.7	87.1	1.1	91.1	0.9	152.7	95.7
median	38.2	49.9	87.1	1.	91.7	0.8	134.	96.4
Quantile .1	19.2	32.	77.6	0.7	76.6	0.6	58.7	77.4
Quantile .9	85.2	65.3	94.9	1.5	101.4	1.3	279.1	111.4
39	20	0						
length	50							

peak +4	R0	$1/\theta_2$	inf	inflog	newly	newlylog	Reff	AttackRate
mean	52.8	59.	124.4	0.4	245.3	0.5	1111.7	97.3
median	49.	60.3	85.2	0.4	191.5	0.4	787.5	95.8
Quantile .1	23.4	37.8	66.	0.2	73.	0.3	323.7	78.4
Quantile .9	83.	77.6	187.2	0.7	476.9	0.6	2453.5	112.1
43	21	0						
length	50							

peak +8	R0	$1/\theta_2$	inf	inflog	newly	newlylog	Reff	AttackRate
mean	46.9	48.6	1156.2	0.7	2261.1	0.9	1780.1	90.9
median	43.1	44.4	436.3	0.6	1209.1	0.8	1582.6	89.2
Quantile .1	20.6	25.2	94.8	0.2	333.4	0.5	310.7	72.
Quantile .9	82.7	69.5	2478.2	1.1	4392.2	1.3	3442.2	111.6
37	18	0						
length	50							
